# An application of (4YSZ)_0.93_(Fe_2_O_3_)_0.07_ in limiting current oxygen sensor

**DOI:** 10.1038/s41598-019-42266-y

**Published:** 2019-04-05

**Authors:** Xiangnan Wang, Tao Liu, Jingkun Yu

**Affiliations:** School of Metallurgy, Northeastern University, Shenyang, Liaoning 110819 China

## Abstract

(4YSZ)_0.93_(Fe_2_O_3_)_0.07_ and 9 mol% Y_2_O_3_ stabilized ZrO_2_ (9YSZ) were synthesized by co-precipitation method and their crystalline structure, microstructure, electronic conductivity, total conductivity were characterized. A limiting current oxygen sensor was assembled with (4YSZ)_0.93_(Fe_2_O_3_)_0.07_ dense diffusion barrier and 9YSZ solid electrolyte by Pt sintered-paste method. Influences of temperature (*T*), oxygen concentration (*x*(O_2_)) and water vapor pressure (*p*(H_2_O)) on sensing characteristics of the limiting current oxygen sensor were investigated. The crystalline structure of (4YSZ)_0.93_(Fe_2_O_3_)_0.07_ and 9YSZ belong to cubic structure with ${\bf{Fm}}\overline{{\bf{3}}}{\bf{m}}$. The total conductivity of (4YSZ)_0.93_(Fe_2_O_3_)_0.07_ is higher than that of 9YSZ and the electronic and total conductivities of the samples meet the linear relationship with 1000/*T*. The limiting current oxygen sensor exhibits excellent sensing characteristics under test conditions. The effects of *T*, *x*(O_2_) and *p*(H_2_O) are as follows: Log(*I*_L_·*T*) depends linearly on 1000/*T*, *I*_L_ depends linearly on *x*(O_2_) and *I*_L_ is not significantly dependent on *p*(H_2_O).

## Introduction

It is essential to control the air-fuel ratio (A/F) to increase fuel economy and decrease environmental pollution since industrial furnaces and automobile industry expend immense amounts of energy. Solid electrolyte oxygen sensors play an important role for detecting the oxygen concentration in exhaust gas. Solid electrolyte oxygen sensors include two types: galvanic cell type and limiting current type. Potential of galvanic cell oxygen sensor is a function of logarithm of oxygen concentration ratio, causing the sensor to be less sensitive at high oxygen concentration. Limiting current of limiting current oxygen sensors is directly a linear function of oxygen concentration, resulting in the measurement range to be higher accuracy^1^. Limiting current oxygen sensors consist of three types: aperture type, porous type and dense diffusion barrier type. Early researches focused on aperture and porous types limiting current oxygen sensors, but unfortunately, there are some problems, such as pores are easily blocked and porous porosity is difficult to control. These weaknesses restrict the application of aperture and porous types limiting current oxygen sensors. In contrast, limiting current oxygen sensor with mixed ionic-electronic conducting oxides (MIECs) as dense diffusion barrier has been widely concerned by overcoming the shortcomings of the two previous limiting current oxygen sensors^2^. At the same time, the study of MIECs has also attracted the attention of researchers due to its widely application in gas sensors, solid oxide fuel cell and oxygen separation membranes at high temperatures^3–5^. YSZ is an excellent solid electrolyte due to its outstanding chemical stability, physical stability and oxide ionic conductivity^6–8^. Many researches were conducted on dense diffusion barrier limiting current oxygen sensor with different MIECs as dense diffusion barrier and YSZ solid electrolyte^9–12^. Peng *et al*.^9^ developed a sensor with Pt-YSZ composite as dense diffusion barrier. The results showed that the sensor had a limiting current plateau for *x*(O_2_) up to 6% and the *I*_L_ depended linearly on *x*(O_2_). Xia *et al*.^10^ developed a sensor with La_0.8_Sr_0.2_MnO_3_ (LSM) as dense diffusion barrier by Pt sintered-paste method. The results showed that the method can avoid the mismatch of shrinkage between two kinds of LSM and YSZ materials during sintering and can provide a limiting current plateau in a full range of air-fuel ratio. Shi *et al*.^11^ developed a sensor with LSM-YSZ composite as dense diffusion barrier by co-pressing and co-sintering method. The results showed that the sensor exhibited a quite low operating temperature. At 400 °C, the limiting current plateau appeared in voltages of 0.7–1.2 V and it showed a good linear relationship with *x*(O_2_) up to 10%. Wang *et al*.^12^ developed a sensor with La_0.8_Sr_0.2_FeO_3_ (LSF) as dense diffusion barrier. The results showed that the sensor exhibited a performance in oxygen concentrations range of 0–21%. In the above researches, YSZ is solid electrolyte, while the dense diffusion barrier materials have the problem of high cost or mismatch with YSZ solid electrolyte. Fe_2_O_3_ is a transition metal oxide and Fe^3+^ will redox at different oxygen concentration to form redox Fe^3+^/Fe^2+^ couple. Doping Fe_2_O_3_ into YSZ can make the material have electronic and ionic conductivity. On the other hand, Fe_2_O_3_ doped YSZ has good chemical compatibility with YSZ solid electrolyte at elevated temperature. The research on doping Fe_2_O_3_ into YSZ to get MIEC as dense diffusion barrier has not reported yet.

In this paper, (4YSZ)_0.93_(Fe_2_O_3_)_0.07_ and 9YSZ were synthesized by co-precipitation method and their crystalline structure, microstructure, electronic conductivity, total conductivity were characterized, respectively. A limiting current oxygen sensor with (4YSZ)_0.93_(Fe_2_O_3_)_0.07_ as dense diffusion barrier and 9YSZ solid electrolyte was developed by Pt sintered-paste method. The effects of *T*, *x*(O_2_) and *p*(H_2_O) were studied, respectively.

## Experimental

**Figure 1:**
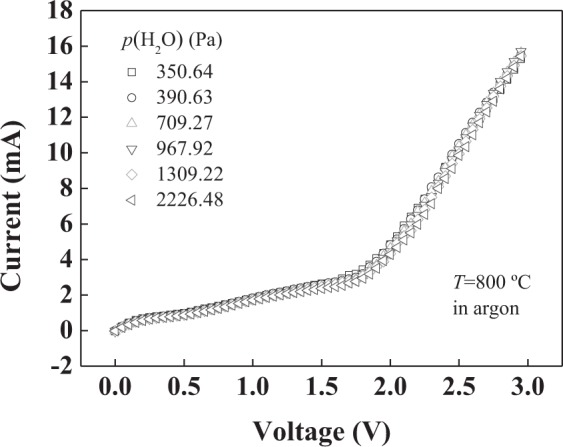
The cross section of dense diffusion barrier limiting current oxygen sensor.

(4YSZ)_0.93_(Fe_2_O_3_)_0.07_ and 9YSZ powders were synthesized by co-precipitation method. The analytical reagents Y(NO_3_)_3_·6H_2_O (purity 99.99%), ZrOCl_2_·8H_2_O (purity 99.9%), FeCl_3_·6H_2_O (purity 99%) and NH_3_·H_2_O (purity 0.1 M) were purchased from Aladdin (www.aladdin-e.com) and used directly without further purification. First, reagents Y(NO_3_)_3_·6H_2_O, ZrOCl_2_·8H_2_O and FeCl_3_·6H_2_O were weighed according to stoichiometry and dissolved in homemade distilled water with intense stirring. Second, reagent NH_3_·H_2_O was dripped into the salt solution with continuous stirring until pH reached 9 to get precipitates. The precipitates were washed by distilled water and ethanol for two times, respectively, and then dried at 70 °C for 12 h to obtain precursor powders. Thirdly, the precursor powders were calcined at 800 °C for 12 h to obtain solid solution powders. Crystalline structure of the solid solution powders was characterized by X-ray diffraction (Philips 3040/60, Netherlands, www.usa.philips.com, Cu Kα, 40 mA, 40 kV) and the data were refined and calculated by GSAS software^13^. We pressed the solid solution powders and then sintered it at 1600 °C for 10 h in air to obtain the samples for measurement of electrical conductivity, microstructure and sensors. Electronic and total conductivities were measured by Hebb-Wagner and DC van der Pauw methods, respectively. Microstructure of the samples on the cross-section was observed by scanning electron microscopy (Ultra Plus, Germany, www.zeiss.com.cn). A limiting current oxygen sensor was prepared by Pt sintered-paste method with (4YSZ)_0.93_(Fe_2_O_3_)_0.07_ dense diffusion barrier and 9YSZ solid electrolyte. Pt paste (www.guyou01.com.cn) was screen printed on the top and bottom of the 9YSZ solid electrolyte as an electrode, Pt wire (Diameter 0.1 mm, China, www.reagent.com.cn) connected to the electrode as a lead. The sintered (4YSZ)_0.93_(Fe_2_O_3_)_0.07_ sample was attached to the top of the sintered 9YSZ sample with Pt paste and heated at 800 °C for 1 h to obtain a dense diffusion barrier limiting current oxygen sensor. The Pt layer between (4YSZ)_0.93_(Fe_2_O_3_)_0.07_ and 9YSZ is to connect them together and the sides of the sample were sealed with glass glaze (www.sic.ac.cn). The cross section of the sensor is shown in Fig. 1 (not to scale).

**Figure 2:**
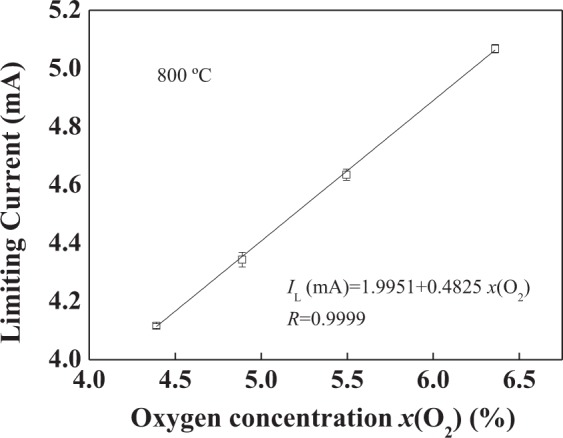
Diagram of testing system for oxygen sensor.

The effects of *T*, *x*(O_2_) and *p*(H_2_O) on the *I-V* characteristics of the sensor were studied, and data were measured by an electrochemical analysis system (LK98B II, China, lanlike.b2b.hc360.com). Limiting current plateau was obtained at temperatures in the order of 800–650 °C and in the oxygen concentration region of 2–7%. By adjusting the temperature of the LiCl·H_2_O saturated solution to control the water vapor pressure in the volumetric flask, argon (Ar) was introduced into the solution to carry the water vapor into the measured furnace to obtain testing conditions of *p*(H_2_O). O_2_ and Ar flow were controlled by capillary flowmeters and the total flow rate in the sensing performance experiment was about 100 ml/min. The testing system mentioned above for oxygen sensor is shown in Fig. 2.

## Results and Discussion

### Property of (4YSZ)_0.93_(Fe_2_O_3_)_0.07_ and 9YSZ

**Figure 3:**
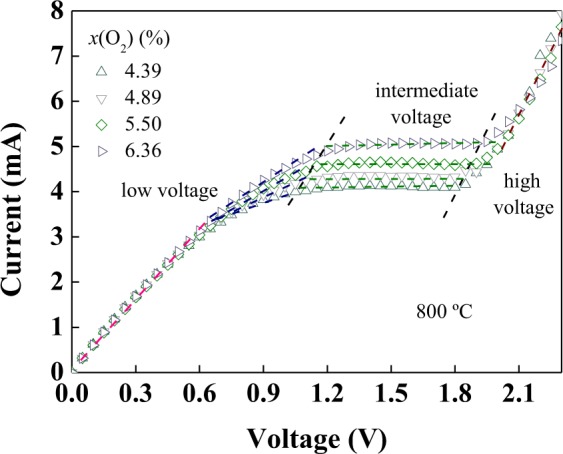
Crystalline structure and calculated cell parameters of the 9YSZ and (4YSZ)_0.93_(Fe_2_O_3_)_0.07_ samples.

The crystalline structure of 9YSZ and (4YSZ)_0.93_(Fe_2_O_3_)_0.07_ belongs to cubic structure with ${\rm{Fm}}\overline{3}{\rm{m}}$ shown in Fig. 3(a), which is consistent with previous studies^14^. The diffraction peaks of monoclinic ZrO_2_ (m-ZrO_2_) in 9YSZ sample are found at 2*θ* of 28.32° and 31.39°, which may be due to incomplete reactions. In the (4YSZ)_0.93_(Fe_2_O_3_)_0.07_ sample, the diffraction peak intensity of the m-ZrO_2_ at 28.32° becomes weaker and the diffraction peak intensity at 31.39° position disappears, which shows that the doping of Fe_2_O_3_ is more beneficial to the formation of cubic ZrO_2_ (c-ZrO_2_). Meanwhile, we did not find the diffraction peak of Fe_2_O_3_ in the sample. For comparison, we further increased the *x* and obtained the (4YSZ)_0.93_(Fe_2_O_3_)_0.07_ sample, and found the diffraction peak of Fe_2_O_3_, which indicating that Fe_2_O_3_ did not doped into YSZ. The calculated cell parameters of samples are shown in Fig. 3(b), it is found that the cell parameters reduced from 5.130209 of the YSZ samples to 5.123664 of the (4YSZ)_0.93_(Fe_2_O_3_)_0.07_ samples and the cell volume reduced from 135.022 to 134.506, indicating that Fe_2_O_3_ is doped into YSZ. This decrease of the cell parameters is due to the substitution of smaller Fe^3+^ ions (0.055 nm) in YSZ for Zr^4+^ ions (0.084 nm)^15,16^.

**Figure 4:**
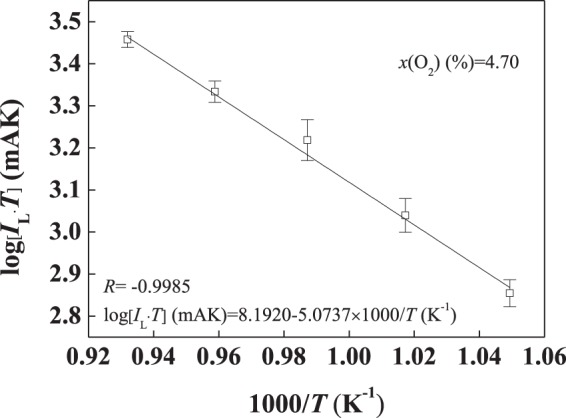
The microstructure of the 9YSZ and (4YSZ)_0.93_(Fe_2_O_3_)_0.07_ samples on the cross-section.

**Figure 5:**
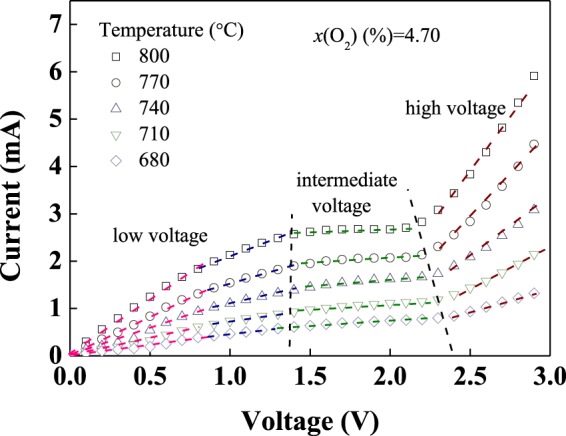
The electrical conductivity of the 9YSZ and (4YSZ)_0.93_(Fe_2_O_3_)_0.07_ samples.

The microstructure of the 9YSZ and (4YSZ)_0.93_(Fe_2_O_3_)_0.07_ samples on the cross-section is shown in Fig. 4. It can be seen from the diagram that the grains of the 9YSZ sample can be seen clearly and evenly distributed, and the grains of the (4YSZ)_0.93_(Fe_2_O_3_)_0.07_ sample are closely linked together, and the samples are dense. The total conductivity of the 9YSZ and (4YSZ)_0.93_(Fe_2_O_3_)_0.07_ samples and the electronic conductivity of (4YSZ)_0.93_(Fe_2_O_3_)_0.07_ sample are shown in Fig. 5. The total conductivity of (4YSZ)_0.93_(Fe_2_O_3_)_0.07_ is higher than that of the YSZ sample. The total and electronic conductivity of the samples meet the linear relationship with the temperature.

### Effect of temperature on the sensor

**Figure 6:**
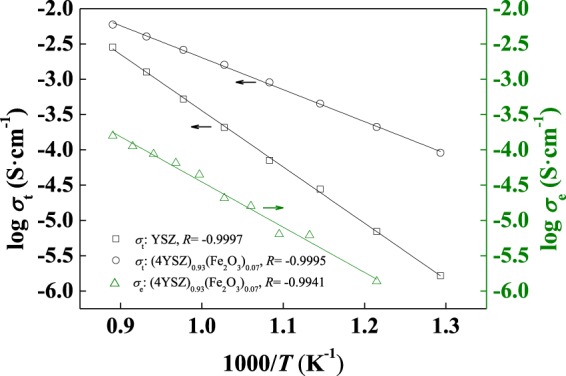
*I*-*V* characteristic curves of the oxygen sensor in different temperature in an oxygen concentration of 4.70%.

*I*-*V* characteristic curves of the oxygen sensor with (4YSZ)_0.93_(Fe_2_O_3_)_0.07_ dense diffusion barrier in the temperature range of 800–680 °C in an oxygen concentration of 4.70% are shown in Fig. 6. It can be seen that from the pattern that the *I*-*V* characteristic curves of the sensor are mainly divided into three regions, which are located in low voltage region, intermediate voltage region and high voltage region, respectively, and the dashed lines representing a linear fit to the data. (1) In low voltage region, output current increases linearly with increasing applied voltage, which is predominantly caused by ohmic behavior of solid electrolyte^17^. Since the conductivity of the solid electrolyte increases with increasing temperature, the slope of the ohmic section at high temperature is higher than that at low temperature. At the end of the region, the slope of ohmic behavior has a slight decrease, which may be in a transitional area. (2) In intermediate voltage region, when the pump oxygen rate of the solid electrolyte is equal to the oxygen diffusion rate of the dense diffusion barrier, the *I*-*V* relationship approaches a limiting current plateau, which is a valuable experimental data. The limiting current *I*_L_ of the sensor increases with increasing *T*, which is due to the increase of conductivity of (4YSZ)_0.93_(Fe_2_O_3_)_0.07_ dense diffusion barrier. (3) In high voltage, output current increases with applied voltage, which is caused by oxide reductions of solid electrolyte^18,19^.

**Figure 7:**
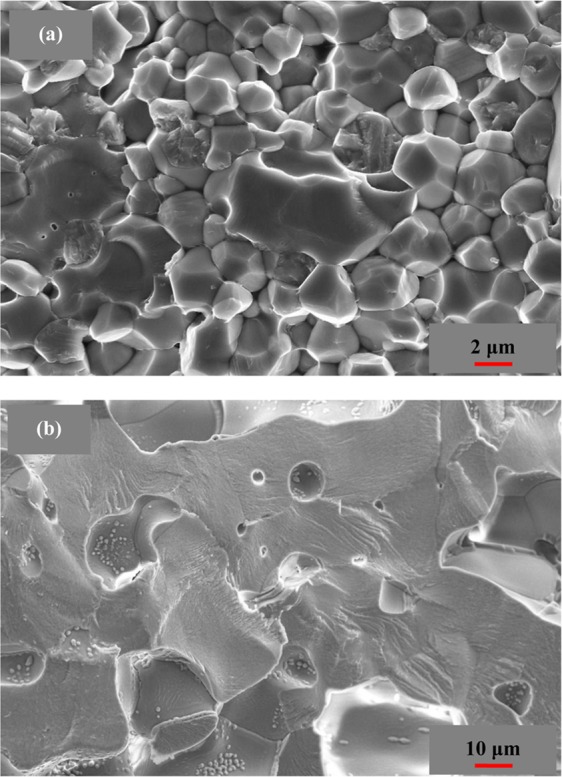
Relationship between log(*I*_L_·*T*) and 1000/*T* in an oxygen concentration of 4.70%.

The relationship between $\mathrm{log}({{I}}_{{\rm{L}}}\cdot {T})$ and 1000/*T* is shown in Fig. 7. The linear correlation coefficient *R* is −0.9985. In addition, the activation energy of the oxygen ion in the (4YSZ)_0.93_(Fe_2_O_3_)_0.07_ dense diffusion barrier can be calculated according to the fitting line, which is 0.9 eV in ref.^20^.

### Effect of oxygen concentration on the sensor

**Figure 8:**
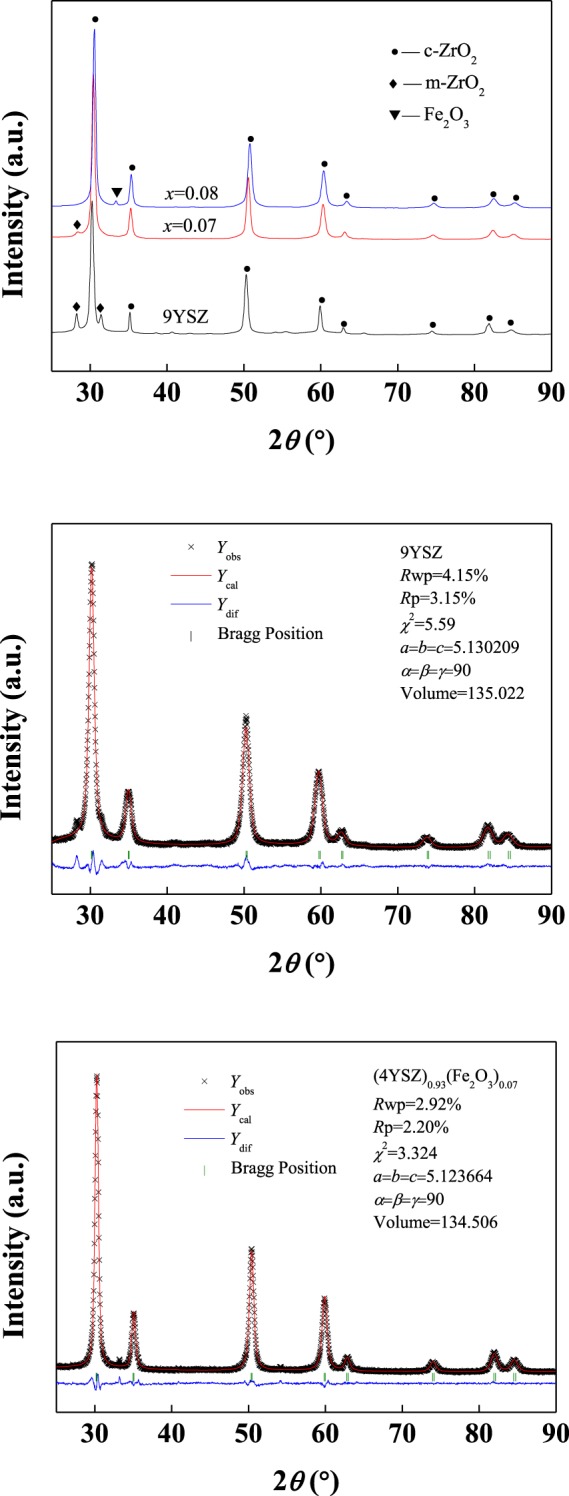
*I*–*V* characteristic curves of the sensor at different oxygen concentrations at 800 °C.

*I*-*V* characteristic curves of the sensor with (YSZ)_0.93_(Fe_2_O_3_)_0.07_ dense diffusion barrier at different oxygen concentrations are shown in Fig. 8. It can be seen that the distinct limiting current plateau is observed at oxygen concentrations of 4.3–6.4%. At high temperatures, oxygen is adsorbed on the outside of the (4YSZ)_0.93_(Fe_2_O_3_)_0.07_ dense diffusion barrier and the adsorbed oxygen forms oxygen ions by absorbing two electrons at the (4YSZ)_0.93_(Fe_2_O_3_)_0.07_/Pt/air three-phase boundary. Oxygen ions transported from the surface of the (4YSZ)_0.93_(Fe_2_O_3_)_0.07_ dense diffusion barrier to the (4YSZ)_0.93_(Fe_2_O_3_)_0.07_/9YSZ interfaces driven by oxygen pressure difference since the oxygen pressure of the surface is higher than that of the interfaces. Oxygen ions lose electrons becoming oxygen molecules, and then release oxygen. Due to the high electronic conductivity and no potential gradient of the (4YSZ)_0.93_(Fe_2_O_3_)_0.07_ dense diffusion barrier, oxygen ions are pressure-diffused through the dense diffusion barrier only by oxygen pressure differential between the two sides of the (4YSZ)_0.93_(Fe_2_O_3_)_0.07_ dense diffusion barrier. The migration rate of oxygen ions from the (4YSZ)_0.93_(Fe_2_O_3_)_0.07_/9YSZ interfaces to the 9YSZ solid electrolyte surface is affected by the applied voltage across solid electrolyte. The pump oxygen current rate increases with increasing applied voltage. When the pump oxygen rate of the 9YSZ solid electrolyte is equal to the oxygen diffusion rate of the (4YSZ)_0.93_(Fe_2_O_3_)_0.07_ dense diffusion barrier and the applied voltage increases to a certain value, the limiting current is obtained. Under lower oxygen concentration, the limiting current plateau of the sensor is obtained at lower voltage, which is consistent with results in refs^21–23^.

**Figure 9:**
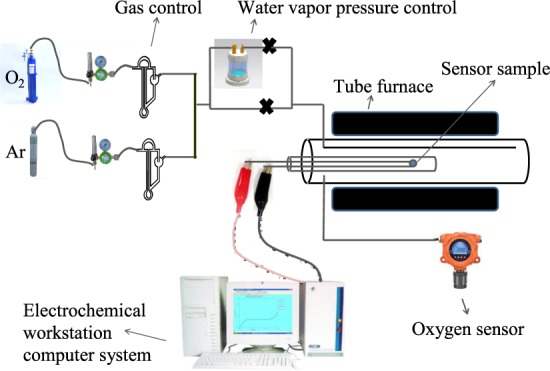
The relationship between the limiting current and oxygen concentration at 800 °C.

The relationship between the limiting current and oxygen concentration at 800 °C is shown in Fig. 9. It can be seen that the limiting current and the oxygen concentration display a good linear relationship, which is related to the correlation theory of the Knudsen diffusion^24^.

1 $${{I}}_{{\rm{L}}}=\frac{4{F}{{D}}_{{\rm{K}}}{SP}}{{RTL}}\cdot {x}({{\rm{O}}}_{2})$$

where *D*_K_, *P*, *T*, *F*, *R*, *S* and *L* are oxygen diffusion coefficient of Knudsen diffusion, partial pressure difference of diffused gas between electrodes I and II, temperature, Faraday constant, gas constant, total cross-sectional area and length of diffusion path, respectively.

The relationship between diffusion coefficient (*D*_T_) and temperature (*T*) for the solid ion diffusion mode of solid theory is as follows:

2 $${{D}}_{{\rm{T}}}={{D}}_{{\rm{O}}}\cdot \exp (-\frac{\varepsilon }{{{k}}_{{\rm{B}}}{T}})$$

where *D*_o_, *ε* and *k*_B_ are constant of the frequency factor, activation energy for the diffusion process and the Boltzmann constant, respectively.

Combining equations (1) and (2) obtains the equation (3):

3 $${I}_{L}=\frac{{4F}{{D}}_{{O}}{SP}}{{RLT}}\cdot x({O}_{2})\cdot \exp (-\varepsilon /{k}_{B}T)$$

If the oxygen concentration is stable, so:

4 $$a=\frac{{4F}{{D}}_{{O}}{SP}}{{RL}}x({O}_{2})$$

Equation (4) is brought into equation (3) to get equation (5):

5 $${I}_{L}={a}\cdot \frac{1}{{T}}\cdot \exp (\,-\,\varepsilon /{k}_{{\rm{B}}}T)$$

Equation (5) becomes a logarithmic function to obtain equation (6):

6 $$\mathrm{log}\,{{I}}_{{\rm{L}}}=A-log\,{T}-\frac{{\varepsilon }}{{{k}}_{{\rm{B}}}{T}}$$

and then,

7 $$B=-\,\frac{\varepsilon }{{k}_{{\rm{B}}}}$$

Now, equation (6) becomes equation (8), and then becomes equation (9):

8 $$\mathrm{log}\,{I}_{{\rm{L}}}={\rm{A}}-\,\mathrm{log}\,T+{\rm{B}}\cdot \frac{1}{{T}}\,$$

9 $$\mathrm{log}\,({I}_{{\rm{L}}}\cdot T)={\rm{A}}+{\rm{B}}\cdot \frac{1}{{T}}\,$$

According to equation (9), $\mathrm{log}({I}_{{\rm{L}}}\cdot T)$ and 1000/*T* have a good linear relationship at different temperatures. As shown in Fig. 8, the linear correlation between $\mathrm{log}({I}_{{\rm{L}}}\cdot T)$ and 1000/*T* is well in accordance with the Knudsen diffusion model.

### Effect of water vapor pressure on the sensor

**Figure 10:**
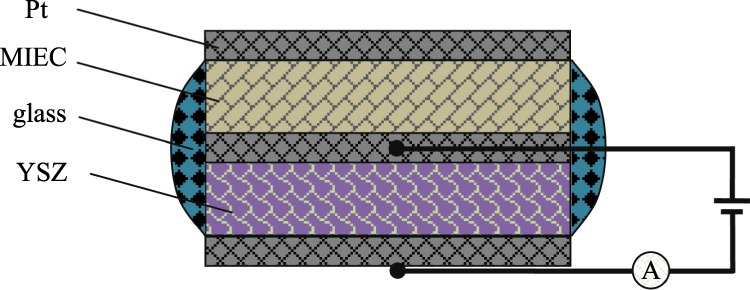
Relationship between *I*–*V* characteristics and water vapor pressure in an argon atmosphere at 800 °C.

The water vapor pressure dependence of the *I*-*V* characteristics at 800 °C is shown in Fig. 10. The water vapor pressure was achieved by passing dry argon through LiCl·H_2_O solution. The relationship between the water vapor pressure above saturated LiCl·H_2_O solution and temperatures of 23.90–54.84 °C is known according to ref.^25^. The temperature was controlled by a constant temperature water bath. As can be seen from the figure, water vapor pressure has no obvious effect on *I*-*V* characteristics of the sensor in the measured region.

## Conclusions

(4YSZ)_0.93_(Fe_2_O_3_)_0.07_ dense diffusion barrier was synthesized by co-precipitation method. The crystalline structure of (4YSZ)_0.93_(Fe_2_O_3_)_0.07_ and 9YSZ belong to cubic structure. The total conductivity of (4YSZ)_0.93_(Fe_2_O_3_)_0.07_ is higher than that of 9YSZ and the electrical conductivity of the samples meet the linear relationship with 1000/*T*. The limiting current oxygen sensor based on (4YSZ)_0.93_(Fe_2_O_3_)_0.07_ dense diffusion barrier and 9YSZ solid electrolyte was developed and it demonstrated a very good linear relationship between log(*I*_L_·*T*) and 1000/*T* well in accordance with the Knudsen diffusion model. At the same time, the sensor exhibits a good sensing performance with the oxygen concentration of 4.39–6.36% at 800 °C, which also agrees with the Knudsen diffusion model. Water vapor pressure in the measured region has no obvious effect on *I*-*V* characteristics of the sensor, which means that the sensor has good selectivity for oxygen.

## Data Availability

The datasets generated during and/or analysed during the current study are available from the corresponding author on reasonable request.
